# Real Time Influenza Monitoring Using Hospital Big Data in Combination with Machine Learning Methods: Comparison Study

**DOI:** 10.2196/11361

**Published:** 2018-12-21

**Authors:** Canelle Poirier, Audrey Lavenu, Valérie Bertaud, Boris Campillo-Gimenez, Emmanuel Chazard, Marc Cuggia, Guillaume Bouzillé

**Affiliations:** 1 Laboratoire Traitement du Signal et de l'Image Université de Rennes 1 Rennes France; 2 INSERM U1099 Rennes France; 3 Centre d'Investigation Clinique de Rennes Université de Rennes 1 Rennes France; 4 Centre Hospitalier Universitaire de Rennes Centre de Données Cliniques Rennes France; 5 Comprehensive Cancer Regional Center Eugene Marquis Rennes France; 6 Centre d'Etudes et de Recherche en Informatique Médicale EA2694 Université de Lille Lille France; 7 Public Health Department Centre Hospitalier Régional Universitaire de Lille Lille France

**Keywords:** electronic health records, big data, infodemiology, infoveillance, influenza, machine learning, Sentinelles network

## Abstract

**Background:**

Traditional surveillance systems produce estimates of influenza-like illness (ILI) incidence rates, but with 1- to 3-week delay. Accurate real-time monitoring systems for influenza outbreaks could be useful for making public health decisions. Several studies have investigated the possibility of using internet users’ activity data and different statistical models to predict influenza epidemics in near real time. However, very few studies have investigated hospital big data.

**Objective:**

Here, we compared internet and electronic health records (EHRs) data and different statistical models to identify the best approach (data type and statistical model) for ILI estimates in real time.

**Methods:**

We used Google data for internet data and the clinical data warehouse eHOP, which included all EHRs from Rennes University Hospital (France), for hospital data. We compared 3 statistical models—random forest, elastic net, and support vector machine (SVM).

**Results:**

For national ILI incidence rate, the best correlation was 0.98 and the mean squared error (MSE) was 866 obtained with hospital data and the SVM model. For the Brittany region, the best correlation was 0.923 and MSE was 2364 obtained with hospital data and the SVM model.

**Conclusions:**

We found that EHR data together with historical epidemiological information (French Sentinelles network) allowed for accurately predicting ILI incidence rates for the entire France as well as for the Brittany region and outperformed the internet data whatever was the statistical model used. Moreover, the performance of the two statistical models, elastic net and SVM, was comparable.

## Introduction

### Background

Influenza is a major public health problem. Outbreaks cause up to 5 million severe cases and 500,000 deaths per year worldwide [1-5]. During influenza peaks, large increase in visits to general practitioners and emergency departments causes health care system disruption.

To reduce its impact and help organize adapted sanitary responses, it is necessary to monitor influenza-like illness (ILI; any acute respiratory infection with fever *≥* 38°C, cough, and onset within the last 10 days) activity. Some countries rely on clinical surveillance schemes based on reports by sentinel physicians [6], where volunteer outpatient health care providers report all ILI cases seen during consultation each week. In France, ILI incidence rate is then computed at the national or regional scale by taking into account the number of sentinel physicians and medical density of the area of interest. ILI surveillance networks produce estimates of ILI incidence rates, but with a 1- to 3-week delay due to the time needed for data processing and aggregation. This time lag is an issue for public health decision making [2,7]. Therefore, there is a growing interest in finding ways to avoid this information gap. Nsoesie et al [8] reviewed methods for influenza forecasting, including temporal series and compartmental methods. The authors showed that these models have limitations. For instance, influenza activity is not consistent from season to season, which is a problem for temporal series. Alternative strategies have been proposed, including using different data sources, such as meteorological or demographic data, combined with ILI surveillance network data [9-11] or big data, particularly Web data [12]. With over 3.2 billion Web users, data flows from the internet are huge and of all types; they can be from social networks (eg, Facebook and Twitter), viewing sites, (eg, YouTube and Netflix), shopping sites, (eg, Amazon and Cdiscount), but also from sales or rentals website between particulars (eg, Craigslist and Airbnb). In the case of influenza, some studies used data from Google [2,4,9,13-16], Twitter [17,18], or Wikipedia [19-21]. The biggest advantage of Web data is that they are produced in real time. One of the first and most famous studies on the use of internet data for detecting influenza epidemics is Google Flu Trends [13,22], a Web service operated by Google. They showed that internet users’ searches are strongly correlated with influenza epidemics. However, for the influenza season 2012-2013, Google Flu Trends clearly overestimated the flu epidemic due to the announcement of a pandemic that increased the internet users’ search frequency, whereas the pandemic finally did not appear. The lack of robustness, due to the sensitivity to the internet users’ behavioral changes and the modifications of the search engine performance led to stop the Google Flu Trends algorithm [2,23,24].

Some authors updated the Google Flu Trends algorithm by including data from other sources, such as historical flu information for instance or temperature [2,13-16]. Yang et al [2] proposed an approach that relies on Web-based data (Centers for Diseases Control ILI activity and Google data) and on a dynamic statistical model based on a least absolute shrinkage and selection operator (LASSO) regression that allows overcoming the aforementioned issues. At the national scale, the correlation between predictions and incidence rates was 0.98.

The internet is not the only data source that can be used to produce information in real time. With the widespread adoption of electronic health records (EHRs), hospitals also produce a huge amount of data that are collected during hospitalization. Moreover, many hospitals are implementing information technology tools to facilitate the access to clinical data for secondary-use purposes. Among these technologies, clinical data warehouses (CDWs) are one of the solutions for hospital big data (HBD) exploitation [25-28]. The most famous is the Informatics for Integrating Biology & the Bedside (i2b2) project, developed by the Harvard Medical School, which is now used worldwide for clinical research [29,30]. In addition, it has been shown that influenza activity changes detected retrospectively with EHR-based ILI indicators are highly correlated with the influenza surveillance data [31,32]. However, few HBD-based models have been developed to monitor influenza [7,33]. Santillana et al proposed a model using HBD and a machine learning algorithm (support vector machine [SVM]) with a good performance at the regional scale [7]. The correlation between estimates and ILI incidence rates ranges from 0.90 to 0.99, depending on the region and season.

### Objectives

It would be interesting to determine whether HBD gives similar, better, or lower results than internet data with these statistical models (machine learning and regression). To this aim, we first evaluated HBD capacity to estimate influenza incidence rates compared with internet data (Google data). Then, we aim to find the best statistical model to estimate influenza incidence rates at the national and regional scales by using HBD or internet data. As these models have been described in the literature, we focused on two machine learning algorithms, random forest (RF) and SVM, and a linear regression model, elastic net.

## Methods

### Data Sources

#### Clinical Data Warehouse eHOP

At Rennes University Hospital (France), we developed our own CDW technology called eHOP. eHOP integrates structured (laboratory test results, prescriptions, and International Classification of Diseases 10th Revision, ICD-10, diagnoses) and unstructured (discharge letter, pathology reports, and operative reports) patients data. It includes data from 1.2 million in- and outpatients and 45 million documents that correspond to 510 million structured elements. eHOP consists of a powerful search engine system that can identify patients with specific criteria by querying unstructured data with keywords, or structured data with querying codes based on terminologies. eHOP is routinely used for clinical research. The first approach to obtain eHOP data connected with ILI was to perform different full-text queries to retrieve patients who had, at least, one document in their EHR that matched the following search criteria:

1. Queries directly connected with flu or ILI were as follows:
  - “flu”
  - “flu” or “ILI”
  - “flu” or “ILI”, in the absence of “flu vaccination”
  - “flu vaccination”
  - “flu” or “ILI”, only in emergency department reports
2. Queries connected with flu symptoms were as follows:
  - “fever” or “pyrexia”
  - “body aches” or “muscular pain”
  - “fever or pyrexia” or “body aches or muscular pain”
  - “flu vaccination”
  - “fever or pyrexia” and “body aches or muscular pain”
3. Drug query was as follows:
  - “Tamiflu”

The second approach was to leverage structured data with the support of appropriate terminologies:

1. ICD-10 queries were as follows: J09.x, J10.x, or J11.x (chapters corresponding to influenza in ICD-10). We retained all diagnosis-related groups with these codes.
2. Laboratory queries were as follows: influenza testing by reverse transcription polymerase chain reaction; we retained test reports with positive or negative results because the aim was to evaluate more generally ILI symptom fluctuations and not specifically influenza.

In total, we did 34 queries. For each query, the eHOP search engine returned all documents containing the chosen keywords (often, several documents for 1 patient and 1 stay). For query aggregation, we kept the oldest document for 1 patient and 1 stay and then calculated, for each week, the number of stays with, at least, one document mentioning the keyword contained in the query. In this way, we obtained 34 variables from the CDW eHOP. Multimedia Appendix 1 shows the queries and the number of concerned stays. We retrieved retrospective data for the period going from December 14, 2003 to October 24, 2016. This study was approved by the local Ethics Committee of Rennes Academic Hospital (approval number 16.69).

#### Google Data

For comparison with internet data, we obtained the frequency per week of the 100 most correlated internet queries (Multimedia Appendices 2 and 3) by French users from Google Correlate [34], and we used this information to retrieve Google Trends data. Unlike Google Correlate, Google Trends data [35] are available in real time, but we had to use Google Correlate to identify the most correlated queries to a signal. The times series passed into Google Correlate are the national flu time series and the regional flu time series (Brittany region) obtained from the French Sentinelles network (see below). The time period used to calculate the correlation is from January 2004 to October 2016. We used the R package gtrendsR to obtain automatically Google Trends data from January 4, 2004 to October 24, 2016 [36,37].

#### Sentinelles Network Data

We obtained the national (Metropolitan France) and regional (Brittany region, because Rennes University Hospital, from which EHR data were obtained, is situated in this region) ILI incidence rates (per 100,000 inhabitants) from the French Sentinelles network [38-40] from December 28, 2002 to October 24, 2016. We considered these data as the gold standard and used them as independent historical variables for our models.

### Data Preparation

**Figure 1:**
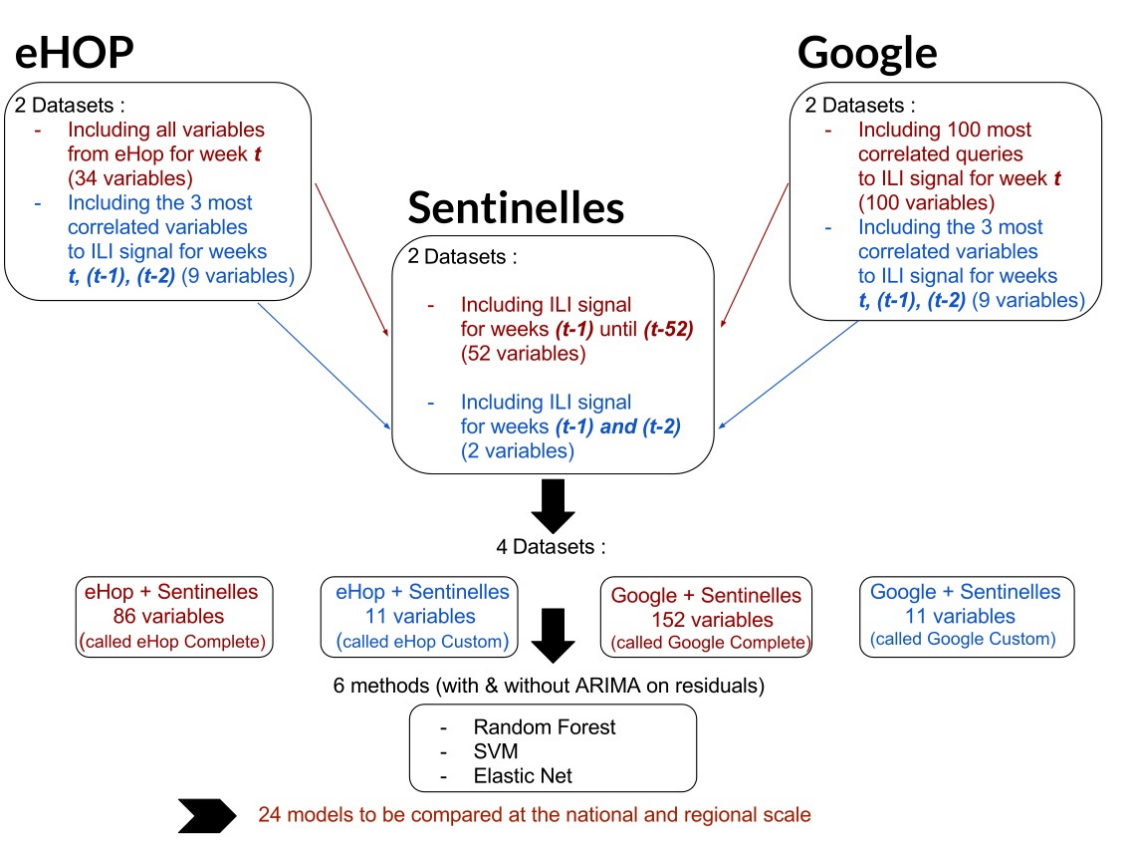
Schematic representation of the study design, including the data preparation and data modeling steps. ILI: influenza-like illness; SVM: support vector machine; ARIMA: autoregressive integrated moving average.

Based on previous studies that included datasets with very different numbers of explanatory variables according to the used statistical model [2,7], we built two datasets (one with a large number of variables and another with a reduced number of selected variables) from eHOP and Google data, for both the national and regional analyses (Figure 1).

Each one of these four datasets was completed with historical Sentinelles data. Therefore, for this study, we used the following:

1. eHOP Complete: this eHOP dataset included all variables from eHOP and the historical data from the Sentinelles network with the ILI estimates for the 52 weeks that preceded the week under study (thus, from t-1 to t-52).
2. eHOP Custom: this eHOP dataset included the 3 most correlated variables between January 2004 and October 2016 from eHOP for the ILI signal for week t, −1 (t-1), and −2 (t-2), and historical information from the Sentinelles network with ILI estimates for t-1 and t-2.
3. Google Complete: this Google dataset included the 100 most ILI activity-correlated queries from Google Trends and historical information from the Sentinelles network with ILI estimates for t-1 to t-52.
4. Google Custom: this Google dataset included the 3 most ILI activity-correlated queries between January 2004 and October 2016 from Google Trends for t, (t-1), and (t-2) and historical data from the Sentinelles network with ILI estimates for (t-1) and (t-2).

### Statistical Models

Our test period started on December 28, 2009 and finished on October 24, 2016. We fitted our models using a training dataset that corresponded to the data for the previous 6 years. Each model was dynamically recalibrated every week to incorporate new information. For instance, to estimate the ILI activity fluctuations for the week starting on December 28, 2009, the training data consisted of data from December 21, 2003 to December 21, 2009.

#### Elastic Net

Elastic net is a regularized regression method that takes into account the correlation between explanatory variables and also a large number of predictors [41]. It combines the penalties of the LASSO and Ridge methods, thus allowing keeping the advantages of both methods and overcoming their limitations [42,43]. With datasets that may have up to 152 potentially correlated variables, we performed the elastic net regression analysis using the R package glmnet and the associated functions [36,44]. We fixed a coefficient alpha equal to.5 to give the same importance to the LASSO and Ridge constraints. We optimized the shrinkage parameter lambda via a 10-fold cross validation.

#### Random Forest

RF model combines decision trees constructed at training time using the general bootstrap aggregating technique (known as bagging) [45]. We used the R package randomForest to create RF models with a number of decision trees equal to 1500 [36,46].

#### Support Vector Machine

SVM is a supervised machine learning algorithm that can be used for classification or regression analyses [47]. Unlike multivariate regression models, SVM can learn nonlinear functions with the kernel trick that maps independent variables in a higher dimensional feature space. As Santillana et al [7], we used the linear kernel and optimized the cost parameter via a 10-fold cross validation with the R package e1071 [36,48].

### Validity

Elastic net is a model that fulfills some assumptions on residuals. Means and variances must be constant, and residuals must be not correlated. Thus, residuals are called white noise. To test the stationarity and whiteness, we used Dickey Fuller’s and Box-Pierce’s tests available from the R packages tseries and stats [36,49]. When assumptions were not respected, we fitted residuals with a model of temporal series, called autoregressive integrated moving average (ARIMA) model. For RF and SVM, assumptions on residuals are not required. However, for comparison purpose, we tested them with the ARIMA model on residuals (Multimedia Appendices 4 and 5). We also assessed the calibration of the models by plotting the estimates against the real observations and by adding the regression line [50] (Multimedia Appendices 6 and 7).

### Evaluation

We compared our ILI estimates with ILI incidence rates from the Sentinelles network by calculating different indicators. The mean squared error (MSE); Pearson correlation coefficient (PCC); variation in the height of the epidemic peak (∆H), which corresponds to the difference between the height of the ILI incidence rate peak during the epidemic period estimated by the models and the height estimated by the Sentinelles network; and prediction lag (∆L), which corresponds to the time difference between the ILI incidence rate peak estimated by the models and the peak estimated by the Sentinelles network, were calcuated. For the global comparison (ie, the entire study period), we calculated only the MSE and PCC. We calculated the four metrics only for the epidemic periods (plus 2 weeks before the start and after the end of the epidemic). The start and end date of epidemics were obtained from the Sentinelles network [39]. Indeed, clinicians want to know when an epidemic starts and finishes, as well as its amplitude and severity. Therefore, interepidemic periods are less important. We also calculated the mean of each indicator for each influenza season to assess the model robustness. We also added two indicators to the mean of (∆H) and (∆L): the mean of |∆H| and |∆L|. We used the mean of (∆H) to assess whether the models tended to underestimate or overestimate the peak calculated by the Sentinelles network, and the mean of (∆L) to determine whether the predictions made by our models were too late or too in advance relative to the Sentinelles data. The mean of |∆H| and |∆L| allowed us to assess the estimate variability.

## Results

### Principal Results

Here, we show the results we obtained with the four datasets and three models—RF, SVM, and elastic net+residuals fitted by ARIMA (ElasticNet+ARIMA). The model on residuals was required to fulfill the assumptions for elastic net but not for the RF and SVM models. All results are presented in Multimedia Appendices 4 and 5. Moreover, we present two influenza outbreaks, including the 2010-2011 season (flu outbreak period for which the best estimates were obtained with all models) and the 2013-2014 season (flu outbreak period for which the worst estimates were obtained with all models; Multimedia Appendix 8). The calibration plots are in presented in Multimedia Appendices 7 and 9.

### National Analysis

#### Dataset Comparison

PCC ranged from 0.947 to 0.980 when using the eHOP datasets (Multimedia Appendix 8) and from 0.937 to 0.978 with the Google datasets. MSE ranged from 2292 to 866 for the eHOP and from 2607 to 968 for the Google datasets. The mean PCC values during epidemic periods varied from 0.90 to 0.96 for the eHOP and from 0.87 to 0.96 for the Google datasets. The mean MSE values ranged from 7597 to 2664 for the eHOP and from 9139 to 2805 for the Google datasets.

#### Model Comparison

**Figure 2:**
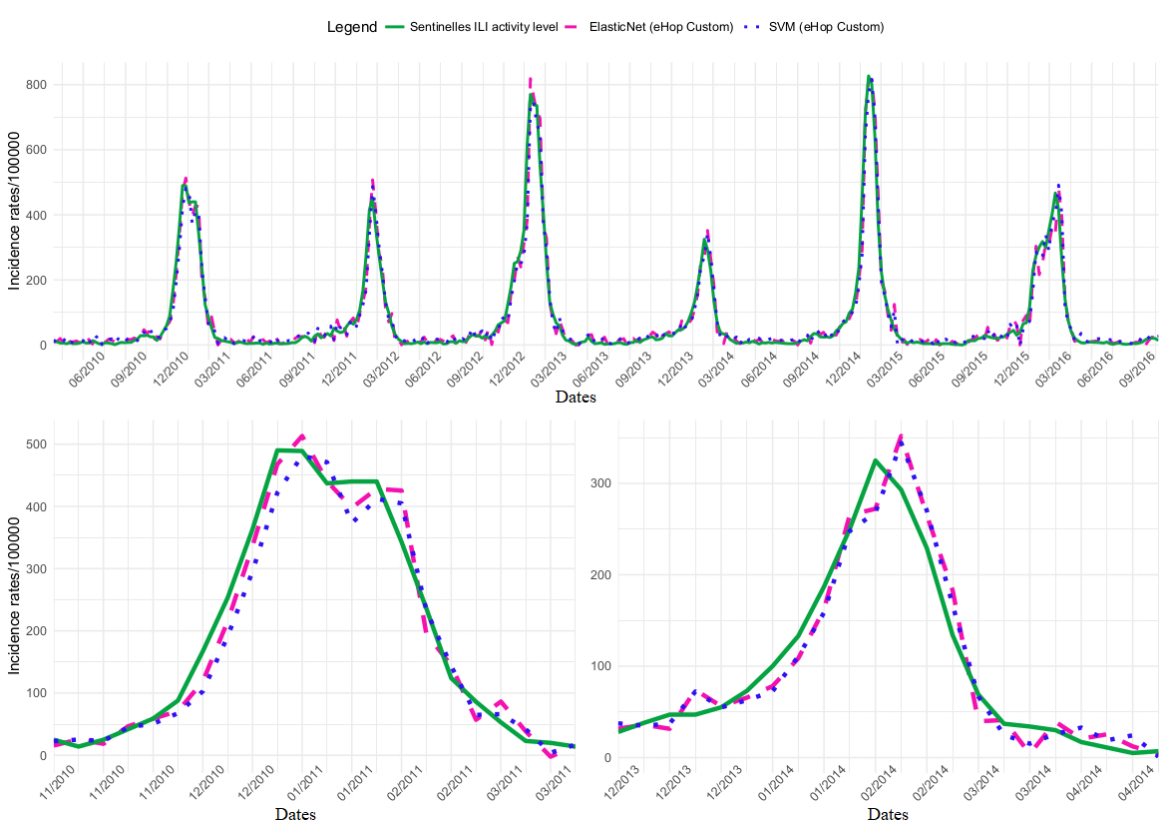
National influenza-like illness (ILI) activity retrospective estimates obtained using the eHOP Custom dataset and the elastic net model with residuals fitted or the support vector machine model compared with the ILI activity levels from the French national Sentinelles networks. Global signal and 2010-2011 and 2013-2014 outbreaks are presented. SVM: support vector machine.

The eHOP Custom dataset gave the best results with the SVM model and ElasticNet+ARIMA (Multimedia Appendix 8). The SVM model and ElasticNet+ARIMA showed similar performance concerning the global activity (PCC=0.98; MSE, <900) and also during epidemic periods (mean values), although PCC decreased (0.96) and the MSE increased (*>* 2500). Both models tended to overestimate the height of the epidemic peaks (∆H=6 with SVM; ∆H=26 with ElasticNet+ARIMA), but the SVM model was slightly more accurate (|∆H|=19 for SVM; |∆H|=30 for the ElasticNet+ARIMA model). Conversely, the SVM model showed a larger prediction lag (∆L=+0.83). Figure 2 illustrates the estimates obtained with the best models (SVM and ElasticNet+ARIMA with the dataset eHop Custom).

The same figure with the dataset Google Custom is presented in Multimedia Appendix 10. In the same way, there is a figure with eHOP Custom and Google Custom datasets with the model ElasticNet+ARIMA presented in Multimedia Appendix 11.

For the outbreak of 2010-2011, eHOP Custom using ElasticNet+ARIMA gave the best PCC (0.98) and the best MSE (1222). With this model, there was a slight overestimation of the height of the epidemic peak (∆H=23) and a prediction lag of 1 week. For the 2013-2014 outbreak, eHOP Custom using SVM gave the best PCC (0.95) and MSE (996), as well as the best ∆H (19) and prediction lag (1 week; Multimedia Appendix 8).

### Regional Analysis

**Figure 3:**
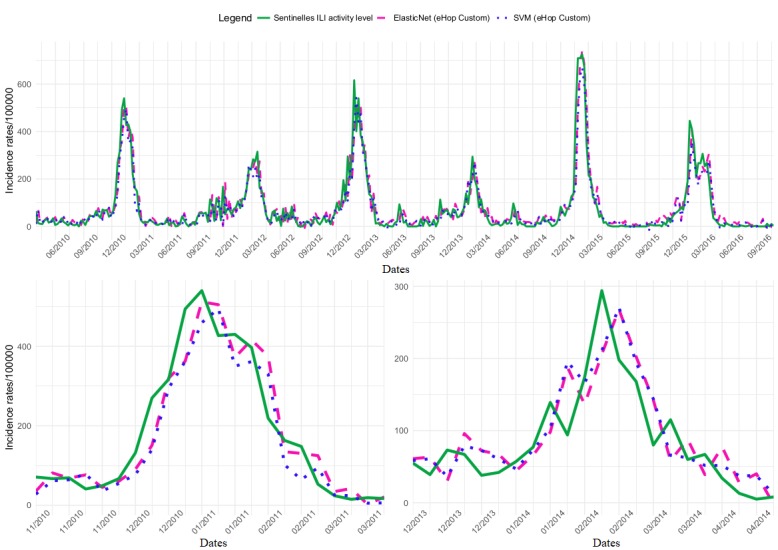
Regional influenza-like illness (ILI) activity retrospective estimates obtained using the eHOP Custom dataset and the elastic net model with residuals fitted or the support vector machine model compared with the ILI activity levels from the French regional Sentinelles networks. Global signal and 2010-2011 and 2013-2014 outbreaks are presented. SVM: support vector machine.

Figure 3 shows that ILI incidence rate variations were more important at the regional than the national level. For this reason, PCC decreased and MSE increased by the order of magnitude. The same figure with the dataset Google Custom is presented in Multimedia Appendix 12.

#### Dataset Comparison

PCC ranged from 0.911 to 0.923 (Multimedia Appendix 8) with the eHOP and from 0.890 to 0.912 with the Google datasets. MSE varied from 2906 to 2364 and from 3348 to 2736 for the eHOP and Google datasets, respectively. During epidemic periods, the mean PCC value ranged from 0.83 to 0.86 and from 0.70 to 0.83 for the eHOP and Google datasets, respectively. The mean MSE values ranged from 7423 to 5893 for the eHOP and from 9598 to 7122 for the Google datasets.

#### Model Comparison

Like at the national scale, eHOP Custom allowed obtaining the best PCC and MSE, and the SVM (PCC=0.923; MSE=2364) and ElasticNet+ARIMA (PCC=0.918; MSE=2451) models showed similar performances (Multimedia Appendix 8). Similar results were obtained also for the mean values during epidemic periods. Nevertheless, the PCC decreased (0.86 for SVM and 0.84 for ElasticNet+ARIMA), and the MSE increased (6050 for SVM and 5999 for ElasticNet+ARIMA). Both models tended to underestimate the height of the epidemic peaks (∆H=−60 with SVM; ∆H=−32 with ElasticNet+ARIMA). The SVM model gave better PCC and MSE than the ElasticNet+ARIMA model, but ElasticNet+ARIMA was slightly more accurate for the epidemic peak height (|∆H|=60 for SVM; |∆H|=38 for the ElasticNet+ARIMA model). Although both models had a prediction lag (∆L=+0.3), the ElasticNet+ARIMA model absolute lag value was smaller than that of SVM (|∆L|=0.7; |∆L|=1). For the 2010-2011 outbreak, eHOP Complete using the RF model gave the best PCC (0.92) and MSE (4263); with this model, there was a slight peak underestimation (∆H=−40) but no prediction lag. For the 2013-2014 epidemic, the best PCC (0.78) and MSE (2113) were obtained with the Google Complete dataset and the ElasticNet+ARIMA model; there was a slight epidemic peak height underestimation (∆H=−26) and 1 week of prediction lag.

## Discussion

### Data

Here, we show that HBD in combination with flu activity-level data from a national surveillance network allows accurately predicting ILI incidence rate at the national and regional scale and outperform Google data in most cases. The correlation coefficients obtained for the French data are comparable to those reported by studies on US data [2,7]. At the national and regional level, the best PCC and the best MSE during the entire study period or during epidemics were obtained using the eHOP Custom dataset. Moreover, the PCC and MSE values obtained with the eHOP datasets were better than those obtained with the Google datasets, particularly at the regional level (PCC 0.911-0.923 vs 0.890-0.912; MSE 2906-2364 vs 3348-2736, respectively; Multimedia Appendix 8). However, the national signal is smoother and less noisy than the regional signal; the contribution of other data sources, such as hospital data or Web data, in addition to historical influenza data is more important at the regional level (Multimedia Appendices 4 and 5). The contribution of these external sources being less important at the national level, the differences observed between hospital data and Web data at this scale could be more significant.

Like internet data, some HBD can be obtained in near real time, especially records from emergency departments that are available on the same day or the day after. This is the most important data source for our models using eHOP datasets. Some other data, such as laboratory results, are available only on a weekly basis; however, they are not the most important data source for our models.

Moreover, in comparison to internet data, HBD have some additional advantages. First, data extracted from CDWs are real health data can give information that cannot be extracted from internet data, particularly information about patients (sex, age, and comorbidities) [51]. In addition, an important clinical aspect is to determine the epidemic severity. With HBD, it is possible to gauge this parameter by taking into account the number of patients who were admitted in intensive care or died as the result of flu. Second, some CDW data (particularly emergency department discharge summaries and laboratory test results) can confirm that people were really affected by influenza or ILI symptoms. On the other hand, people can make internet queries not because they are ill, but for other people, for prevention purposes or just because it is a topical subject. Third, HBD could also be used to estimate the incidence rates of diseases that do not generate internet activity (eg, diseases without or with little media coverage or that are not considered interesting by the general population). Fourth, there is a spatial decorrelation between internet data and the regional estimates that were not observed with the eHOP data. It is quite reasonable that hospital-based data give a better estimate of regional epidemics, although currently, we have only data from Rennes University Hospital that might not be representative of the entire Brittany region.

A major HBD limitation is that, generally, clinical data are not publicly available. In our case, we could only access the Rennes University Hospital HBD. However, the epidemic peak in Brittany could have occurred earlier or later relative to the national peak, and this could have introduced a bias in our estimation. We can hypothesize that ILI estimates, particularly nationwide, might be improved if we could extract information from HBD in other regions. In the United States, a patient research system allows aggregating patient observations from a large number of hospitals in a uniform way [52]. In France, several initiatives have been developed to create search systems. For instance, an ongoing project (Réseau interrégional des Centres de Données Cliniques) [53] in the Northwest area of France associates six University Hospital centers (Angers, Brest, Nantes, Poitiers, and Rennes et Tours) and Orleans Regional Hospital Centre, thus collecting data on patients in the Bretagne, Centre-Val de Loire, and Pays de la Loire regions. This corresponds to 15.5% of Metropolitan France and 14.4% of the entire French population. Another way to aggregate patient data could be a cloud-based platform, and we are currently setting up this kind of architecture; this platform will integrate two University Hospital centers, Brest and Rennes, the French health reimbursement database (Système national d'information interrégimes de l'Assurance Maladie) and registries, such as the birth defect registry or cancer registry.

### Statistical Models

Regarding the statistical models, we show that SVM and elastic net with ARIMA model are fairly comparable with PCC ranging from 0.970 to 0.980 at the national scale and from 0.890 to 0.923 at the regional scale. The SVM and elastic net models in combination with the eHOP custom dataset were the most robust models, although they did not always give the best results. Indeed, they showed the best performance in term of PCC and MSE for the global signal and also for the mean values. Nevertheless, these models have some limits. The main limitation of the SVM model is the very slow parameter optimization when there are many variables. With the SVM model, it can be important to preselect the important variables to reduce the dataset size to improve the optimization speed. For this, one needs a good knowledge of the available data, which may be difficult when using big data. On the other hand, elastic net shows good performance with many variables, which is an advantage when the most relevant variables to estimate ILI incidence rates are not known in advance. The elastic net model is a parametric model that fulfills certain assumptions on residuals, differently from the SVM model. With elastic net, residuals must be fitted to have a statistically valid model. Nevertheless, if we had to choose a model, we would prefer SVM with the eHOP Custom dataset because it has a better PCC than elastic net at the regional scale.

Another limitation is that indicators are better for the global period than for epidemic periods. This implies that models are less efficient during flu outbreaks, while clinical concerns are higher during epidemics when good estimates of the outbreak starting date, amplitude, and end are needed.

Finally, the results of our models with Web data may have been overestimated due to the way we obtained data from Google Correlate. Indeed, Google Correlate used information that we did not have at the beginning of our test period. The time period for our time series passed into Google Correlate is from January 2004 to October 2016. But, the beginning of our test period for our models is January 2010. To be more precise, we should recalculate the correlation coefficients for each week to predict with the data available at that time.

In the same way, to custom datasets, we calculated the 3 most correlated variables on a time period including our test period. To compare the results, we built another dataset from eHOP, including the 3 most correlated variables to ILI regional signal between December 2003 and December 2009 (before our test period), and we applied an ElasticNet+ARIMA model. In this way, we kept 2 variables on the 3 present in the eHOP custom dataset. The difference does not seem significant (Multimedia Appendix 6), but it would be interesting to test this hypothesis with all models at the national and regional scale with Google and eHOP custom datasets.

### Perspectives

Future research could address clinical issues not only nationally or regionally but also at finer spatial resolutions such as a city like Lu et al did [54], a health care institution or in subpopulations. Indeed, by predicting epidemics, it will be possible to organize hospitals during epidemics (eg, bed planning and anticipating overcrowding). Moreover, in this study, we compared internet and HBD data; however, hybrid systems could be developed to take advantage of multiple sources [55,56]. For instance, internet data might avoid the limit of the local source linked to the choice or availability of HBD. Data collected by volunteers who self-report symptoms in near real time could be exploited [57]. Similarly, by combining models, we could retain the benefits of each of them and improve the estimates of ILI incidence rates. For example, we could use another algorithm, such as stacking [58], to concomitantly use the SVM and elastic net models. We could also test other kernels than the linear kernel for SVM models. Finally, we carried out a retrospective study using various models with clinical data in combination with the flu activity from the Sentinelles network to estimate ILI incidence rates in real time. Our models need now to be tested to determine whether they can anticipate and predict ILI incidence rates.

### Conclusions

Here, we showed that HBD is a data source that allows predicting the ILI activity as well or even better than internet data. This can be done using two types of models with similar performance—SVM (a machine learning model) and elastic net (a model of regularized regression). This is a promising way for monitoring ILI incidence rates at the national and local levels. HBD presents several advantages compared with internet data. First, they are real health data and can give information about patients (sex, age, and comorbidities). This could allow for making predictions on ILI activity targeted to a specific group of people. Second, hospital data can be used to determine the epidemic severity by taking into account the number of patients who were admitted in intensive care or died as a result of flu. Third, hospital data (particularly the emergency department discharge summaries and laboratory test results) can confirm that people were really affected by influenza. Finally, HBD could also be used to estimate the incidence rates of diseases that do not generate internet activity. Although massive data cannot take the place of traditional influenza surveillance methods at this time, they could be used to complete them. For instance, real-time forecasting is necessary for decision making. It can also be used to manage the patients’ flow in general practitioners’ offices and hospitals, particularly emergency departments.

## Appendix

Multimedia Appendix 1 eHOP queries (with the number of concerned hospital stays from 2003 to 2016).

Multimedia Appendix 2 The 100 most correlated Google queries at national level.

Multimedia Appendix 3 The 100 most correlated Google queries at regional level.

Multimedia Appendix 4 Accuracy metrics for all seasons obtained with all models for the national scale.

Multimedia Appendix 5 Accuracy metrics for all seasons obtained with all models for the regional scale.

Multimedia Appendix 6 Comparison between two datasets with ElasticNet + ARIMA model: Dataset 1 corresponds to the dataset called eHOP Custom used in the paper and including the 3 most correlated variables to ILI signal between December 2009 to October 2016 (our test period). Dataset 2 includes the 3 most correlated variables to ILI signal between December 2003 to December 2009 (before our test period).

Multimedia Appendix 7 National calibration.

Multimedia Appendix 8 Accuracy metrics for the 2010-2011 (flu outbreak period for which the best estimates were obtained with all models) and 2013-2014 (flu outbreak period for which the worst estimates were obtained with all models) seasons. PCC and MSE for the global period (Global) and mean values (Means) of all indicators for each model during the epidemic periods. In bold, the best results for each dataset. a. Data for the whole France. b. Data for the Brittany region.

Multimedia Appendix 9 Regional calibration.

Multimedia Appendix 10 National ILI activity retrospective estimates obtained using the Google Custom dataset and the Elastic Net model with residuals fitted (pink dashed line) or the SVM model (blue dotted line) compared with the ILI activity levels from the French national Sentinelles networks (green solid line). a. Global signal. b. 2010-2011 and c. 2013-2014 outbreaks.

Multimedia Appendix 11 National ILI activity retrospective estimates obtained using the Google Custom dataset and the Elastic Net model (blue dotted line) or eHOP Custom dataset and the Elastic Net model (pink dashed line) compared with the ILI activity levels from the French national Sentinelles networks (green solid line). a. Global signal. b. 2010-2011 and c. 2013-2014 outbreaks.

Multimedia Appendix 12 Regional ILI activity retrospective estimates obtained using the Google Custom dataset and the Elastic Net model with residuals fitted (pink dashed line) or the SVM model (blue dotted line) compared with the ILI activity levels from the French regional Sentinelles networks (green solid line). a. Global signal. b. 2010-2011 and c. 2013-2014 outbreaks.

## Abbreviations

ARIMA: autoregressive integrated moving average
CDW: clinical data warehouse
EHR: electronic health record
HBD: hospital big data
ILI: influenza-like illness
LASSO: least absolute shrinkage and selection operator
MSE: mean squared error
PCC: Pearson correlation coefficient
RF: random forest
SVM: support vector machine
∆H: epidemic peak
∆L: prediction lag
