# Supplementary material for: Real Time Influenza Monitoring Using Hospital Big Data in Combination with Machine Learning Methods: Comparison Study
Source: JMIR Public Health Surveill. 2018 Dec 21;4(4):e11361. doi: 10.2196/11361 (PMC6320394; doi:10.2196/11361)
Supplement: Multimedia Appendix 1 [file publichealth_v4i4e11361_app1.pdf]

Multimedia Appendix 1 : eHOP queries (with the number of concerned hospital stays from 2003 to 2016)

Full text queries :

1. flu (20 135)
2. flu without vaccine (16 893)
3. flu vaccine (3 317)
4. fever (118 671)
5. aches (8 584)
6. fever or aches (123 332)
7. fever and aches (3 923)
8. fever or aches or flu (132 504)
9. fever and aches or flu (22 405)
10. fever or aches or flu without vaccine (130 254)
11. fever and aches or flu without vaccine (19 275)
12. fever or flu (128 124)
13. aches or flu (26 785)
14. fever or flu without vaccine (125 842)
15. aches or flu without vaccine (23 687)
16. flu in the emergency room (9 886)
17. Tamiflu (1 899)

Appropriate terminologies :

18-23. ICD-10 queries (16 504)
24-34. Test laboratories (6 805)
