# Supplementary material for: Real Time Influenza Monitoring Using Hospital Big Data in Combination with Machine Learning Methods: Comparison Study
Source: JMIR Public Health Surveill. 2018 Dec 21;4(4):e11361. doi: 10.2196/11361 (PMC6320394; doi:10.2196/11361)
Supplement: Multimedia Appendix 2 [file publichealth_v4i4e11361_app2.pdf]

1. grippe incubation
2. grippe contagion
3. incubation grippe
4. epidemie de grippe
5. grippe symptome
6. grippe symptomes
7. epidemie grippe
8. épidémie de grippe
9. contagion grippe
10. épidémie grippe
11. grippe traitement
12. traitement de la grippe
13. comment soigner la grippe
14. soigner grippe
15. symptome de la grippe
16. soigner une grippe
17. grippe en France
18. symptomes de la grippe
19. ski en mars
20. calendrier fevrier
21. grippe carte
22. symptome grippe
23. mois de février
24. la grippe en france
25. mois de fevrier
26. skiset
27. lispach
28. oursinade
29. made in angers
30. date mardi gras
31. chaine thermale
32. ancelle
33. chaine thermale du soleil
34. costume de carnaval
35. fête du mimosa
36. syndrome grippal
37. salon peche
38. fete du mimosa
39. calendrier février
40. joue du loup
41. banh chung
42. la joue
43. le mois de février
44. chastreix
45. confiture oranges
46. la joue du loup
47. grippe epidemie
48. greolières
49. fete des citrons menton
50. orange amere
51. les jouvencelles
52. salon de la peche
53. location ski lyon
54. masque de carnaval
55. esf
56. météo serre chevalier
57. ski set
58. grippe bébé
59. recette de bugnes
60. recette des bugnes
61. oursinades
62. la bresse lispach
63. etat grippal
64. saint jean montclar
65. recette bugne
66. meteo les angles
67. vacance de fevrier
68. sentinelle grippe
69. albiez
70. fete des citrons
71. date de la saint valentin
72. fête des citrons à menton
73. minable le pingouin
74. melezes
75. gresse
76. coloriage carnaval
77. tarif location de ski
78. les melezes
79. oranges ameres
80. carnaval de cadiz
81. dhg
82. printemps ete
83. costume carnaval
84. saint leger les melezes
85. le mont dore
86. gresse en vercors
87. date carnaval
88. le mourtis
89. masque carnaval
90. oranges amères
91. maternelle carnaval
92. vacances de fevrier
93. de carnaval
94. carnaval de limoux
95. ski laguiole
96. fevrier
97. chabanon
98. grippe enceinte
99. montclar
100. bettex
