# Supplementary material for: Real Time Influenza Monitoring Using Hospital Big Data in Combination with Machine Learning Methods: Comparison Study
Source: JMIR Public Health Surveill. 2018 Dec 21;4(4):e11361. doi: 10.2196/11361 (PMC6320394; doi:10.2196/11361)
Supplement: Multimedia Appendix 3 [file publichealth_v4i4e11361_app3.pdf]

1. soigner la grippe
2. grippe contagion
3. grippe symptomes
4. epidemie de grippe
5. grippe incubation
6. grippe symptome
7. traitement de la grippe
8. epidemie grippe
9. grippe traitement
10. état grippal
11. symptome de la grippe
12. grossesse et grippe
13. symptomes de la grippe
14. soigner une grippe
15. épidémie grippe
16. grippe en france
17. comment soigner la grippe
18. symptomes grippe
19. soigner grippe
20. symptome grippe
21. la grippe en france
22. chaine thermale
23. chaine thermale du soleil
24. made in angers
25. incubation de la grippe
26. traitement grippe
27. chaines thermales du soleil
28. costume de carnaval
29. salon peche
30. oursinade
31. lispach
32. salon de la peche
33. skiset
34. confiture oranges
35. oranges ameres
36. confiture orange
37. ski en mars
38. mois de février
39. chastreix
40. texte faire part mariage
41. mois de fevrier
42. fête du mimosa
43. fete des citrons menton
44. location ski lyon
45. ancelle
46. calendrier fevrier
47. fetes des citrons
48. mont dore
49. sentinelle grippe
50. la bresse lispach
51. www.vacaf.org
52. costume carnaval
53. etat grippal
54. les jouvencelles
55. ski esf
56. confiture orange amere
57. ski laguiole
58. minable le pingouin
59. esf
60. joue du loup
61. la joue du loup
62. recette de bugne
63. grippe france
64. la joue
65. printemps ete
66. infosup clermont
67. chaines thermales
68. météo risoul
69. bal de carnaval
70. ski mont dore
71. les mourtis
72. fete du mimosa
73. fete du citron a menton
74. orange amere
75. masque de carnaval
76. fete des citrons
77. collection printemps ete
78. jour de la st valentin
79. orange amère
80. saint jean montclar
81. meteo egypte
82. le mont dore
83. carnaval de limoux
84. recette des bugnes
85. grippal
86. coloriage carnaval
87. meteo metabief
88. nuit de neige
89. météo chatel
90. meteo orcieres
91. date mardi gras
92. vacances février paris
93. grippe intestinale
94. superdevoluy
95. affiche carnaval
96. ski manigod
97. chabanon
98. esf la bresse
99. fête des citrons à menton
100. sentiweb
