# Supplementary material for: Real Time Influenza Monitoring Using Hospital Big Data in Combination with Machine Learning Methods: Comparison Study
Source: JMIR Public Health Surveill. 2018 Dec 21;4(4):e11361. doi: 10.2196/11361 (PMC6320394; doi:10.2196/11361)
Supplement: Multimedia Appendix 4 [file publichealth_v4i4e11361_app4.pdf]

NATIONAL	2010-2011				2011-2012				2012-2013				2013-2014			
	**PCC**	**MSE**	**ΔH**	**ΔL**	**PCC**	**MSE**	**ΔH**	**ΔL**	**PCC**	**MSE**	**ΔH**	**ΔL**	**PCC**	**MSE**	**ΔH**	**ΔL**
**eHOP Custom**																
RF	0.95	4119	50	2	0.88	6447	68	0	0.90	10904	-63	3	0.86	3664	30	1
RF+Arima	0.98	1174	42	1	0.96	1928	79	0	0.95	6128	105	0	0.91	2212	75	1
SVM	0.97	1932	-8	1	0.95	1877	35	0	0.96	4930	-21	1	0.95	996	19	1
SVM+Arima	0.97	1436	23	1	0.97	1450	53	0	0.96	4876	39	0	0.94	1217	43	1
ElasticNet	0.97	1855	4	1	0.92	3056	12	0	0.95	6343	-12	1	0.92	1447	-6	1
Elastic+Arima	0.98	1222	23	1	0.96	1735	56	0	0.96	5102	48	0	0.95	1145	27	1
**Google Custom**																
RF	0.89	6476	-112	5	0.86	4849	-44	1	0.88	15642	-152	1	0.87	2651	27	1
RF+Arima	0.96	1966	-35	0	0.96	1376	-9	0	0.94	7171	83	1	0.93	1347	67	1
SVM	0.96	2815	16	1	0.95	1971	7	0	0.96	5803	12	1	0.91	1711	43	1
SVM+Arima	0.96	2311	41	1	0.96	1653	42	0	0.96	5194	36	1	0.90	1958	-36	1
ElasticNet	0.94	3606	-27	1	0.93	2654	-8	0	0.94	7658	12	1	0.88	1981	2	1
Elastic+Arima	0.96	2394	35	1	0.95	1801	45	0	0.95	6108	65	1	0.92	1664	55	1
**eHOP Complete**																
RF	0.96	3121	54	1	0.89	4797	-2	0	0.94	7832	-112	2	0.86	10518	83	-1
RF+Arima	0.97	1917	67	1	0.96	1636	-9	0	0.97	3148	-15	2	0.85	5285	93	1
SVM	0.95	3046	66	4	0.94	2366	22	0	0.96	4646	-33	1	0.94	2234	49	1
SVM+Arima	0.95	3087	66	4	0.94	2158	28	0	0.96	5174	-32	1	0.94	2267	49	1
ElasticNet	0.94	4807	113	0	0.96	1685	17	0	0.94	7762	92	0	0.88	2164	5	1
Elastic+Arima	0.94	7387	254	0	0.97	1732	30	-1	0.94	7807	136	0	0.94	1730	48	1

**Google Complete**																
RF	0.95	2743	23	0	0.94	2674	8	0	0.96	5711	-112	1	0.93	4931	84	1
RF+Arima	0.96	2392	49	2	0.98	1188	58	0	0.97	3391	27	1	0.97	1249	57	1
SVM	0.95	2671	12	3	0.97	3893	-27	0	0.97	3421	-8	0	0.92	1564	56	1
SVM+Arima	0.94	3107	32	1	0.97	931	-28	0	0.97	3608	-30	0	0.92	1641	56	1
ElasticNet	0.90	5392	-49	4	0.95	1975	-11	0	0.95	6142	6	1	0.94	3360	93	1
Elastic+Arima	0.96	2153	6	1	0.94	2260	11	0	0.96	4081	36	1	0.95	2511	85	1
**Historical Variables**																
RF	0.95	4118	52	1	0.87	5796	45	0	0.92	9265	-72	3	0.85	3588	13	1
RF+Arima	0.97	1307	102	1	0.96	1427	7	0	0.97	4116	-45	0	0.93	2052	85	1
SVM	0.96	2202	50	1	0.94	1902	19	0	0.95	5336	1	1	0.94	1068	37	1
SVM+Arima	0.96	2135	49	1	0.95	1739	18	0	0.96	5301	1	1	0.94	1095	37	1
ElasticNet	0.96	2090	29	1	0.94	1909	2	0	0.96	4957	4	1	0.93	1178	19	1
ElasticNet+Arima	0.97	2060	17	1	0.95	1908	2	0	0.96	4957	4	1	0.93	1178	19	1

NATIONAL	2014-2015				2015-2016				Global		Means					
	**PCC**	**MSE**	**ΔH**	**ΔL**	**PCC**	**MSE**	**ΔH**	**ΔL**	**PCC**	**MSE**	**PCC**	**MSE**	**ΔH**	**|ΔH|**	**ΔL**	**|ΔL|**
**eHOP Custom**																
RF	0.94	11606	-167	0	0.87	4754	-51	2	0.947	2292	0.9	6916	-22	72	1.33	1.33
RF+Arima	0.97	4546	-55	0	0.85	6202	58	1	0.974	1227	0.94	3698	51	71	0.5	0.5
SVM	0.98	3750	-11	1	0.94	2809	24	1	0.980	866	0.96	2716	6	19	0.83	0.83
SVM+Arima	0.98	3482	-8	1	0.94	2703	25	1	0.981	819	0.96	2527	29	33	0.66	0.66
ElasticNet	0.96	5638	-43	0	0.90	4125	-34	1	0.974	1133	0.94	3744	-13	18	0.66	0.66
Elastic+Arima	0.98	3333	-13	1	0.91	3448	15	1	0.980	872	0.96	2664	26	30	0.66	0.66
**Google Custom**																
RF	0.86	18706	-133	2	0.87	6507	-40	2	0.937	2607	0.87	9139	-76	94	2	2
RF+Arima	0.95	8316	-15	1	0.92	3730	44	1	0.972	1171	0.94	3984	22	42	0.66	0.66
SVM	0.97	5325	36	0	0.94	2461	10	1	0.977	968	0.95	3348	21	23	0.66	0.66
SVM+Arima	0.97	5135	36	0	0.95	2189	-72	1	0.979	899	0.95	3073	8	38	0.66	0.66
ElasticNet	0.94	9513	-10	1	0.89	4275	-7	1	0.968	1382	0.92	4948	-6	11	0.83	0.83
Elastic+Arima	0.97	5076	27	1	0.93	3069	38	1	0.977	988	0.95	3352	44	44	0.83	0.83
**eHOP Complete**																
RF	0.92	13468	-123	2	0.85	5843	-45	-5	0.954	2148	0.90	7597	-24	75	-0.2	1.5
RF+Arima	0.96	6706	-134	1	0.89	4612	37	1	0.974	1172	0.93	3884	7	64	1	1
SVM	0.96	6146	114	0	0.95	2379	59	1	0.972	1173	0.95	3469	46	57	1.2	1.2
SVM+Arima	0.96	7044	112	0	0.95	2440	60	1	0.971	1247	0.95	3495	47	58	1.2	1.2
ElasticNet	0.94	9899	72	1	0.89	4311	-74	1	0.962	1693	0.93	5105	38	62	0.5	0.5
Elastic+Arima	0.96	6194	120	1	0.93	2951	-19	1	0.970	1427	0.95	4634	95	101	0.33	0.66
**Google Complete**																
RF	0.92	11601	-121	1	0.94	6922	36	1	0.963	1706	0.94	5764	-14	70	0.66	0.66
RF+Arima	0.94	8529	-33	2	0.96	2780	24	1	0.979	917	0.96	3255	30	45	1.2	1.2
SVM	0.98	4148	81	0	0.94	4130	110	1	0.974	1192	0.96	2805	37	49	0.8	0.8
SVM+Arima	0.99	3430	61	0	0.95	3117	64	0	0.976	11310	0.96	2639	26	45	0.3	0.3
ElasticNet	0.96	7128	36	1	0.96	1920	28	1	0.965	1604	0.94	4320	17	37	1.33	0.33
