# Supplementary material for: Real Time Influenza Monitoring Using Hospital Big Data in Combination with Machine Learning Methods: Comparison Study
Source: JMIR Public Health Surveill. 2018 Dec 21;4(4):e11361. doi: 10.2196/11361 (PMC6320394; doi:10.2196/11361)
Supplement: Multimedia Appendix 5 [file publichealth_v4i4e11361_app5.pdf]

REGIONAL	2010-2011				2011-2012				2012-2013				2013-2014			
	**PCC**	**MSE**	**ΔH**	**ΔL**	**PCC**	**MSE**	**ΔH**	**ΔL**	**PCC**	**MSE**	**ΔH**	**ΔL**	**PCC**	**MSE**	**ΔH**	**ΔL**
**eHOP Custom**																
RF	0.91	5796	-29	-1	0.90	1665	4	-4	0.87	7209	-60	1	0.65	4577	-4	1
RF+Arima	0.92	5524	-24	1	0.85	2029	-6	-4	0.83	9785	-59	1	0.58	5039	3	1
SVM	0.92	5502	-53	1	0.89	1533	-53	-1	0.85	7874	-80	1	0.76	2477	-28	1
SVM+Arima	0.92	4721	-40	1	0.87	1548	-39	-1	0.83	8451	-80	1	0.73	2926	-77	1
ElasticNet	0.88	7029	-99	1	0.90	2092	-93	-1	0.82	12232	-226	1	0.76	2154	-81	1
Elastic+Arima	0.92	4689	-28	0	0.82	2146	-26	-1	0.81	9584	-90	1	0.71	2855	-27	1
**Google Custom**																
RF	0.86	7706	-9	0	0.89	1657	17	-3	0.82	10031	3	1	0.70	2887	-46	1
RF+Arima	0.90	6570	37	0	0.78	3141	57	-3	0.73	15320	54	1	0.68	3277	-26	1
SVM	0.91	6010	-71	0	0.84	2102	-54	1	0.70	14528	1	1	0.65	3247	-36	1
SVM+Arima	0.90	6041	-67	0	0.84	2134	-54	1	0.70	14600	4	1	0.64	3270	-36	1
ElasticNet	0.88	9682	-137	1	0.76	4208	-93	1	0.68	17815	-206	1	0.65	3445	-102	1
Elastic+Arima	0.91	5494	-58	0	0.82	2189	-40	1	0.71	14566	-46	1	0.74	2637	-31	1
**eHOP Complete**																
RF	0.92	4263	-40	0	0.89	2222	18	-1	0.88	8525	-46	1	0.65	9735	26	1
RF+Arima	0.93	4036	-30	0	0.90	1984	20	-1	0.89	8218	-51	1	0.53	8530	13	-2
SVM	0.89	7682	69	3	0.87	1637	-7	-1	0.85	7249	-104	3	0.74	4851	24	1
SVM+Arima	0.89	7607	55	3	0.86	1833	-7	-1	0.85	7293	-104	3	0.72	4552	26	1
ElasticNet	0.86	11416	-178	1	0.87	2755	-107	-1	0.81	12443	-220	1	0.71	2771	-100	1
Elastic+Arima	0.90	5740	-2	1	0.80	2393	-27	-1	0.83	8635	-61	3	0.66	3677	7	1
**Google Complete**																
RF	0.92	4650	-80	0	0.90	1423	-8	-3	0.88	6824	-54	1	0.63	5955	-9	2
RF+Arima	0.92	4512	-28	0	0.80	2659	3	-3	0.82	9973	-47	1	0.60	5248	19	1
SVM	0.84	8664	-97	1	0.33	7233	24	-4	0.83	7955	-67	1	0.56	4735	-43	-1
SVM+Arima	0.85	8231	-98	1	0.28	8047	24	-4	0.83	8262	-68	1	0.55	4871	-1	1
ElasticNet	0.85	10735	-148	1	0.60	4090	-97	-3	0.74	15086	-192	1	0.67	3764	-134	1
Elastic+Arima	0.89	6455	-63	1	0.64	3907	-44	-1	0.75	12229	-28	1	0.78	2113	-26	1
**Historical variables**																
RF	0.92	4520	-45	0	0.77	3120	-3	-1	0.82	8352	-90	1	0.44	7277	4	1
RF+Arima	0.93	3574	-28	0	0.73	3701	10	1	0.75	11561	-84	1	0.45	7522	21	2
SVM	0.89	6392	-46	0	0.71	3406	-19	1	0.73	12857	-173	1	0.60	3934	-42	1
SVM+Arima	0.90	6132	-46	0	0.71	3490	-19	1	0.73	12590	-173	1	0.60	3728	-42	1
ElasticNet	0.91	6211	-81	1	0.68	3702	-63	1	0.71	13385	-89	1	0.58	3369	-62	1
Elastic+Arima	0.91	6193	-84	1	0.68	3637	-55	1	0.74	12423	-86	1	0.53	3673	-58	1

REGIONAL	2014-2015				2015-2016				Global		Means					
	**PCC**	**MSE**	**ΔH**	**ΔL**	**PCC**	**MSE**	**ΔH**	**ΔL**	**PCC**	**MSE**	**PCC**	**MSE**	**ΔH**	**|ΔH|**	**ΔL**	**|ΔL|**
**eHOP Custom**																
RF	0.88	18111	-97	1	0.85	4218	-56	1	0.911	2777	0.84	6929	-40	42	-0.2	1.5
RF+Arima	0.85	18236	-15	0	0.90	3181	-2	1	0.910	2807	0.82	7299	-17	18	0	1.3
SVM	0.91	12865	-58	-1	0.83	6052	-90	1	0.923	2364	0.86	6050	-60	60	0.3	1
SVM+Arima	0.91	12718	-30	-1	0.84	5899	-60	1	0.916	2491	0.85	6044	-54	54	0.3	1
ElasticNet	0.90	21569	-215	-1	0.87	6152	-115	1	0.907	3283	0.86	8538	-138	138	0.3	1
Elastic+Arima	0.90	11949	19	0	0.85	4770	-38	1	0.918	2451	0.84	5999	-32	38	0.3	0.7
**Google Custom**																
RF	0.76	27094	-85	1	0.79	8211	-32	7	0.897	3221	0.80	9598	-25	32	1.2	2.2
RF+Arima	0.77	26400	-87	1	0.80	7422	-21	1	0.880	3780	0.78	10355	2	47	1.2	1.2
SVM	0.88	15569	27	0	0.82	5610	-71	1	0.902	2903	0.8	7844	-34	43	0.7	0.7
SVM+Arima	0.88	15529	26	0	0.81	5680	-72	1	0.903	2894	0.8	7876	-33	43	0.7	0.7
ElasticNet	0.86	20276	-125	0	0.76	8064	-148	1	0.891	3887	0.77	10582	-135	135	0.8	0.8
Elastic+Arima	0.90	12748	113	0	0.78	7041	-36	2	0.900	3021	0.81	7446	-16	54	0.8	0.8
**eHOP Complete**																
RF	0.89	15654	-132	0	0.90	4141	-15	1	0.914	2906	0.86	7423	-32	46	0.3	0.7
RF+Arima	0.88	16625	-134	0	0.89	3601	10	1	0.916	2758	0.84	7166	-29	43	-0.2	0.8
SVM	0.88	15132	92	0	0.86	4363	-35	1	0.911	2756	0.85	6819	7	55	1.2	1.5
SVM+Arima	0.88	16229	131	0	0.86	4250	-36	1	0.909	2816	0.84	6961	11	60	1.2	1.5
ElasticNet	0.89	20134	-166	0	0.86	6992	-124	1	0.905	3745	0.83	9419	-149	149	0.5	0.8
Elastic+Arima	0.92	10032	48	0	0.84	4880	-76	1	0.917	2483	0.83	5893	-19	37	0.8	1.2
**Google Complete**																
RF	0.88	17301	-131	1	0.80	6581	-91	3	0.912	2736	0.83	7122	-62	62	0.7	1.7
RF+Arima	0.88	17178	-131	1	0.79	6666	-84	3	0.909	2767	0.80	7706	-45	52	0.5	1.5
SVM	0.87	17624	-83	0	0.76	8609	-45	3	0.89	3348	0.70	9137	-52	59	0	1.7
SVM+Arima	0.87	15552	7	0	0.76	8522	-48	3	0.89	3265	0.69	8914	-31	41	0.3	1.7
ElasticNet	0.87	21226	-168	-1	0.75	7302	-120	3	0.893	3815	0.75	10367	-143	143	0.3	1.7
Elastic+Arima	0.91	11243	6	0	0.77	7488	-28	2	0.903	2967	0.79	7239	-31	32	0.7	1
**Historical variables**																
RF	0.79	31289	-249	2	0.79	8259	79	2	0.876	3627	0.76	10470	-51	78	0.8	1.2
RF+Arima	0.85	20251	-60	0	0.78	10314	129	1	0.885	3432	0.75	9487	-2	55	0.8	0.8
SVM	0.82	25670	-8	0	0.74	8516	7	2	0.876	3578	0.75	10021	-47	49	0.8	0.8
SVM+Arima	0.82	24632	-15	0	0.74	8479	15	2	0.885	3431	0.75	9950	-47	52	0.8	0.8
ElasticNet	0.85	20298	-13	0	0.77	7321	8	1	0.884	3378	0.75	9048	-50	52	0.8	0.8
Elastic+Arima	0.85	20038	4	0	0.77	7361	8	1	0.887	3287	0.75	8887	-45	49	0.8	0.8
