# Supplementary material for: Real Time Influenza Monitoring Using Hospital Big Data in Combination with Machine Learning Methods: Comparison Study
Source: JMIR Public Health Surveill. 2018 Dec 21;4(4):e11361. doi: 10.2196/11361 (PMC6320394; doi:10.2196/11361)
Supplement: Multimedia Appendix 6 [file publichealth_v4i4e11361_app6.pdf]

**REGIONAL**	**2010-2011**				**2011-2012**				**2012-2013**				**2013-2014**			
	**PCC**	**MSE**	**ΔH**	**ΔL**	**PCC**	**MSE**	**ΔH**	**ΔL**	**PCC**	**MSE**	**ΔH**	**ΔL**	**PCC**	**MSE**	**ΔH**	**ΔL**
**eHOP Custom**																
Dataset 1	0.92	4689	-28	0	0.82	2146	-26	-1	0.81	9584	-90	1	0.71	2855	-27	1
Dataset 2	0.91	6298	50	1	0.73	3357	14	-4	0.84	8291	-34	3	0.74	3584	7	1

**REGIONAL**	**2014-2015**				**2015-2016**				**Global**		**Means**					
	**PCC**	**MSE**	**ΔH**	**ΔL**	**PCC**	**MSE**	**ΔH**	**ΔL**	**PCC**	**MSE**	**PCC**	**MSE**	**ΔH**	**|ΔH|**	**ΔL**	**|ΔL|**
**eHOP Custom**																
Dataset 1	0.90	11949	19	0	0.85	4770	-38	1	0.92	2451	0.84	5999	-32	38	0.3	0.7
Dataset 2	0.93	8638	14	0	0.90	3066	40	1	0.92	2347	0.84	5539	16	27	0.3	1.7
