# Supplementary figures and images for: Real Time Influenza Monitoring Using Hospital Big Data in Combination with Machine Learning Methods: Comparison Study
Source: JMIR Public Health Surveill. 2018 Dec 21;4(4):e11361. doi: 10.2196/11361 (PMC6320394; doi:10.2196/11361)

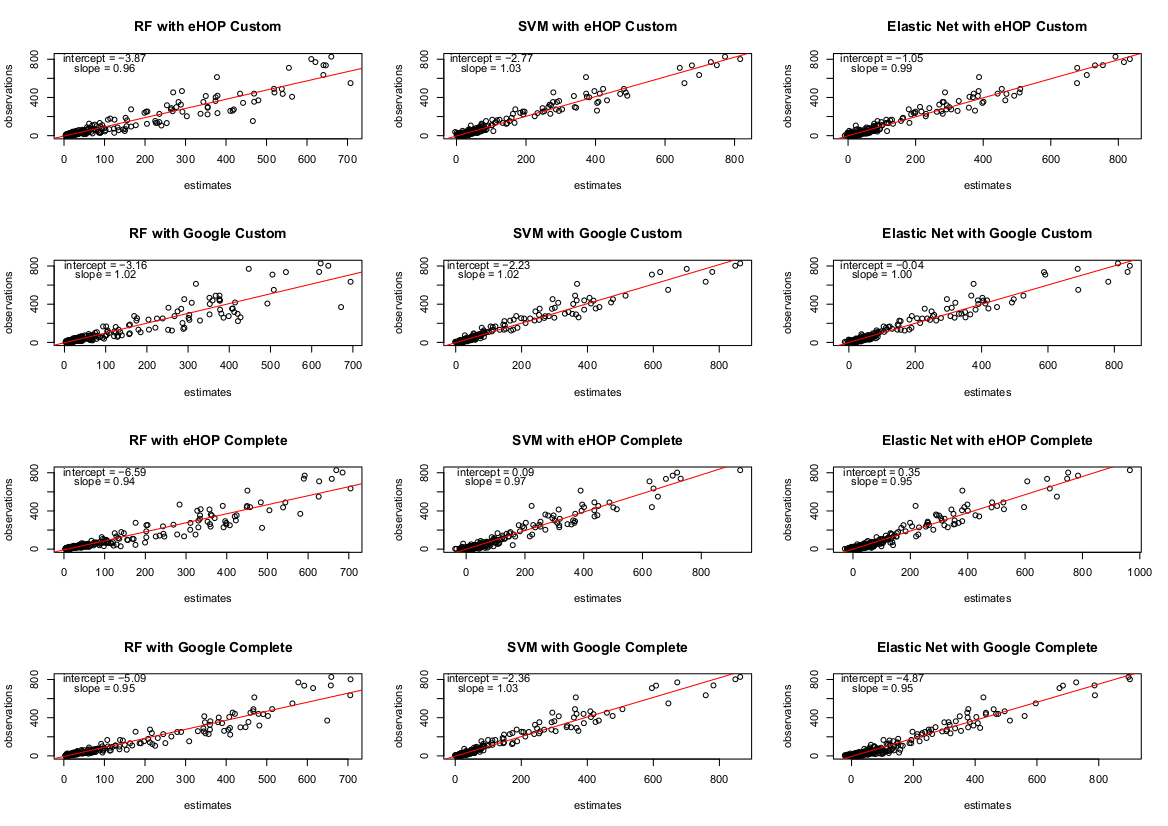

Supplement: Multimedia Appendix 7 [file publichealth_v4i4e11361_app7.png]

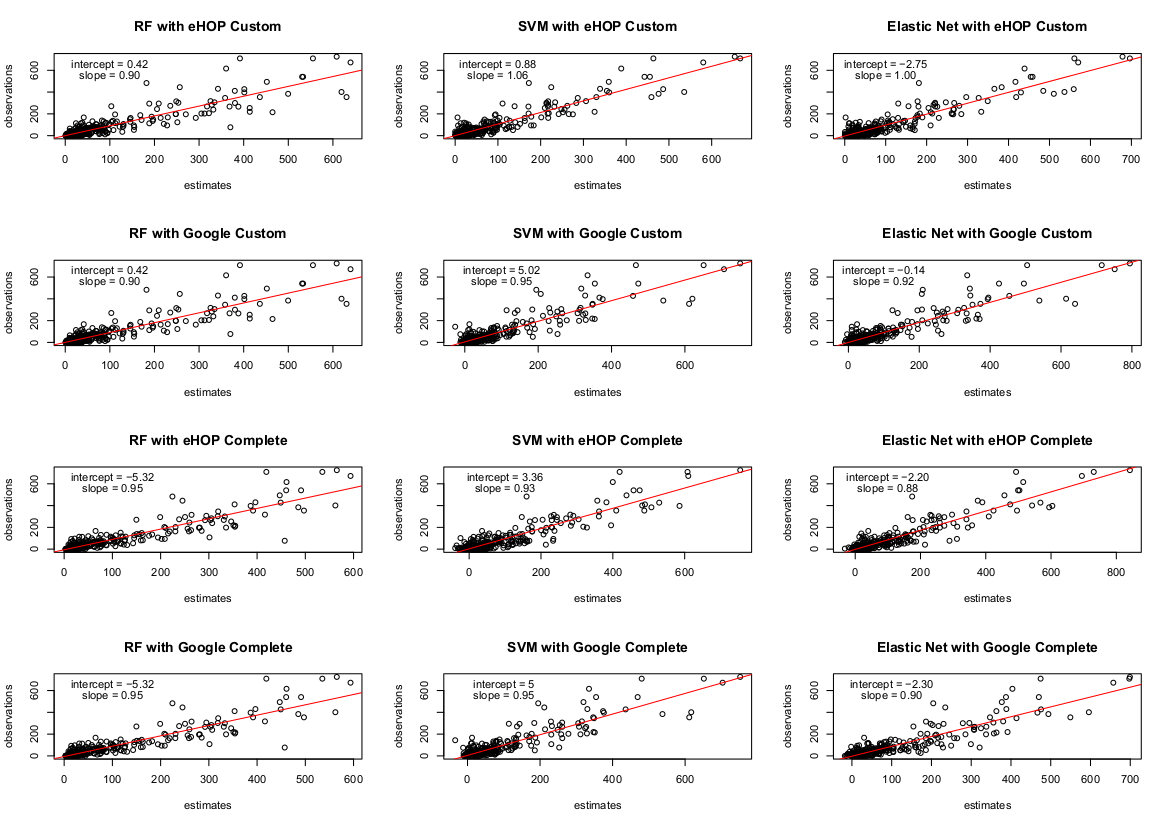

Supplement: Multimedia Appendix 9 [file publichealth_v4i4e11361_app9.png]

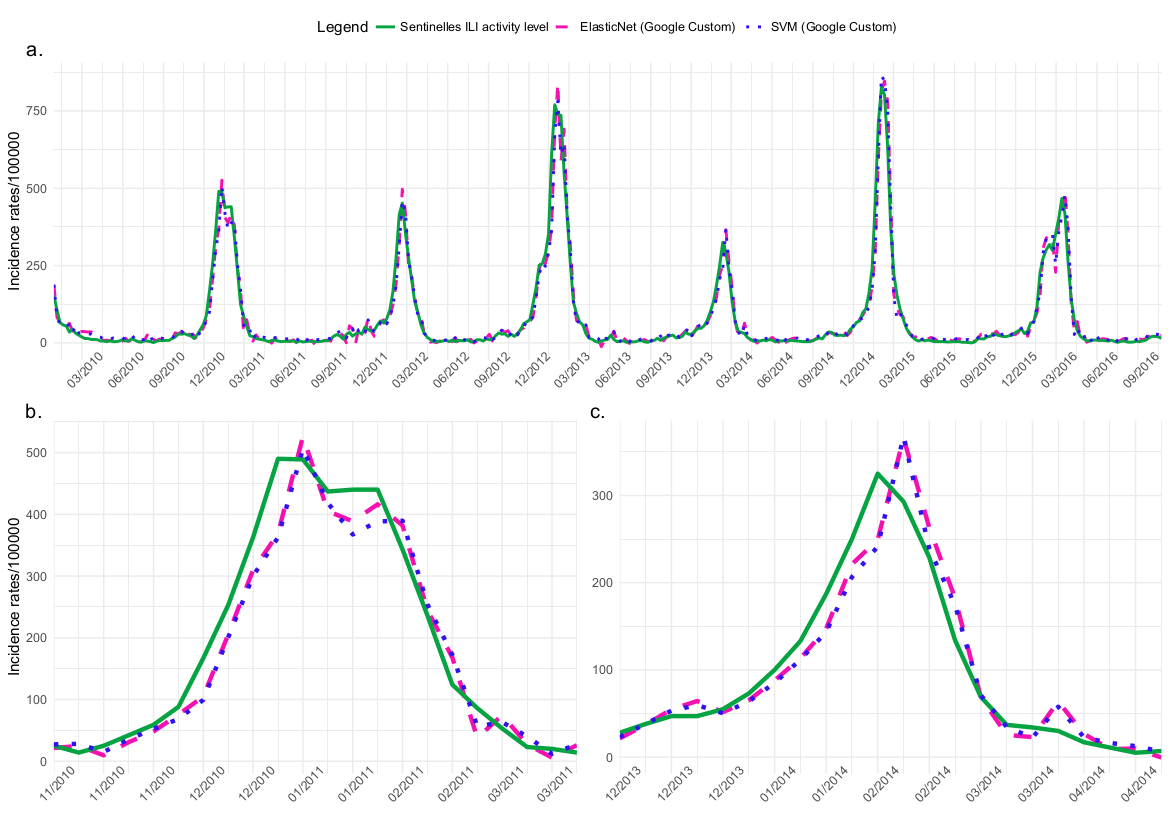

Supplement: Multimedia Appendix 10 [file publichealth_v4i4e11361_app10.png]

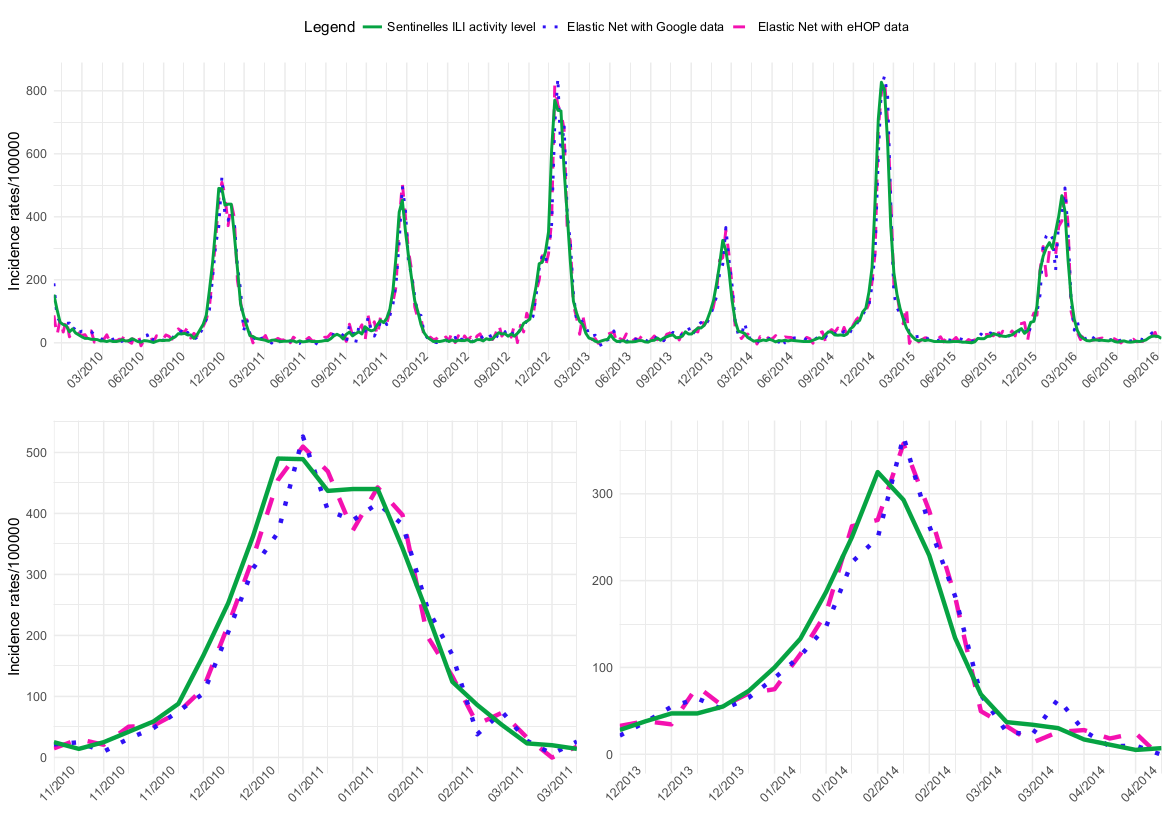

Supplement: Multimedia Appendix 11 [file publichealth_v4i4e11361_app11.png]

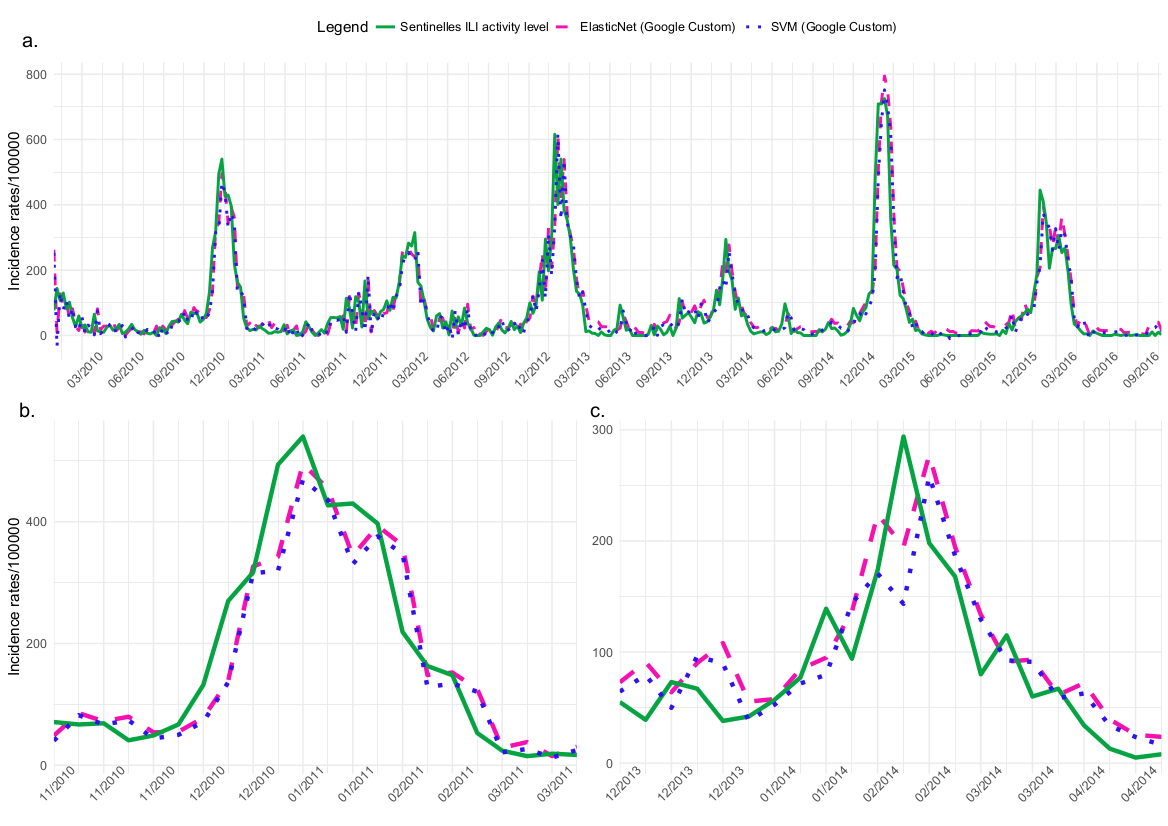

Supplement: Multimedia Appendix 12 [file publichealth_v4i4e11361_app12.png]
