# Supplementary material for: Real Time Influenza Monitoring Using Hospital Big Data in Combination with Machine Learning Methods: Comparison Study
Source: JMIR Public Health Surveill. 2018 Dec 21;4(4):e11361. doi: 10.2196/11361 (PMC6320394; doi:10.2196/11361)
Supplement: Multimedia Appendix 8 [file publichealth_v4i4e11361_app8.pdf]

Multimedia Appendix 8. Accuracy metrics for the 2010-2011 (flu outbreak period for which the best estimates were obtained with all models) and 2013-2014 (flu outbreak period for which the worst estimates were obtained with all models) seasons. PCC and MSE for the global period (Global) and mean values (Means) of all indicators for each model during the epidemic periods. In bold, the best results for each dataset. a. Data for the whole France. b. Data for the Brittany region.

**a. National**	2010-2011				2013-2014				Global		Means					
	PCC	MSE	ΔH	ΔL	PCC	MSE	ΔH	ΔL	PCC	MSE	PCC	MSE	ΔH	|ΔH|	ΔL	|ΔL|
**eHOP Custom**																
RF	0.95	4119	50	2	0.86	3664	30	1	0.947	2292	0.9	6916	–22	72	1.33	1.33
SVM	0.97	1932	–8	1	**0.95**	**996**	19	1	**0.98**	**866**	0.96	2716	6	19	0.83	0.83
Elastic+Arima	**0.98**	**1222**	23	1	0.95	1145	27	1	0.98	872	**0.96**	**2664**	26	30	0.66	0.66
**Google Custom**																
RF	0.89	6476	–112	5	0.87	2651	27	1	0.937	2607	0.87	9139	–76	94	2	2
SVM	0.96	2815	16	1	0.91	1711	43	1	**0.977**	**968**	**0.95**	**3348**	21	23	0.66	0.66
Elastic+Arima	**0.96**	**2394**	35	1	**0.92**	**1664**	55	1	0.977	988	0.95	3352	44	44	0.83	0.83
**eHOP Complete**																
RF	0.96	3121	54	1	0.86	10518	83	–1	0.954	2148	0.9	7597	–24	75	–0.2	1.5
SVM	**0.95**	**3046**	66	4	0.94	2234	49	1	**0.972**	**1173**	**0.95**	**3469**	46	57	1.2	1.2
Elastic+Arima	0.94	7387	254	0	**0.94**	**1730**	48	1	0.97	1427	0.95	4634	95	101	0.33	0.66
**Google Complete**																
RF	0.95	2743	23	0	0.93	4931	84	1	0.963	1706	0.94	5764	–14	70	0.66	0.66
SVM	0.95	2671	12	3	0.92	**1564**	56	1	0.974	1192	**0.96**	**2805**	37	49	0.8	0.8
Elastic+Arima	**0.96**	**2153**	6	1	**0.95**	2511	85	1	**0.978**	**1057**	0.96	2967	39	38	0.66	0.66

**b. Regional**	2010-2011				2013-2014				Global		Means					
	PCC	MSE	ΔH	ΔL	PCC	MSE	ΔH	ΔL	PCC	MSE	PCC	MSE	ΔH	|ΔH|	ΔL	|ΔL|
**eHOP Custom**																
RF	0.91	5796	–29	–1	0.65	4577	–4	1	0.911	2777	0.84	6929	–40	42	–0.2	1.5
SVM	0.92	5502	–53	1	**0.76**	**2477**	–28	1	**0.923**	**2364**	**0.86**	**6050**	–60	60	0.3	1
Elastic+Arima	**0.92**	**4689**	–28	0	0.71	2855	–27	1	0.918	2451	0.84	5999	–32	38	0.3	0.7
**Google Custom**																
RF	0.86	7706	–9	0	0.7	2887	–46	1	0.897	3221	0.80	9598	–25	32	1.2	2.2
SVM	0.91	6010	–71	0	0.65	3247	–36	1	**0.902**	**2903**	0.80	7844	–34	43	0.7	0.7
Elastic+Arima	**0.91**	**5494**	–58	0	**0.74**	**2637**	–31	1	0.9	3021	**0.81**	**7446**	–16	54	0.8	0.8
**eHOP Complete**																
RF	**0.92**	**4263**	–40	0	0.65	9735	26	1	0.914	2906	**0.86**	7423	–32	46	0.3	0.7
SVM	0.89	7682	69	3	**0.74**	**4851**	24	1	0.911	2756	0.85	6819	7	55	1.2	1.5
Elastic+Arima	0.9	5740	–2	1	0.66	3677	7	1	**0.917**	**2483**	0.83	**5893**	–19	37	0.8	1.2
**Google Complete**																
RF	**0.92**	**4650**	–80	0	0.63	5955	–9	2	**0.912**	**2736**	**0.83**	**7122**	–62	62	0.7	1.7
SVM	0.84	8664	–97	1	0.56	4735	–43	–1	0.890	3348	0.70	9137	–52	59	0	1.67
Elastic+Arima	0.89	6455	–63	1	**0.78**	**2113**	–26	1	0.903	2967	0.79	7239	–31	32	0.7	1
